# Breeding *Polyommatus icarus* Serves as a Large-Scale and Environmentally Friendly Source of Precisely Tuned Photonic Nanoarchitectures

**DOI:** 10.3390/insects14080716

**Published:** 2023-08-18

**Authors:** Gábor Piszter, Zsolt Bálint, Krisztián Kertész, Lajos Szatmári, Gábor Sramkó, László Péter Biró

**Affiliations:** 1 Centre for Energy Research, Institute of Technical Physics and Materials Science, Konkoly-Thege Miklós út 29-33, H-1121 Budapest, Hungary; 2 Hungarian Natural History Museum, Baross utca 13, H-1121 Budapest, Hungary; 3 ELKH-DE Conservation Biology Research Group, Egyetem tér 1, H-4032 Debrecen, Hungary; 4 Department of Botany, University of Debrecen, Egyetem tér 1, H-4032 Debrecen, Hungary

**Keywords:** Common Blue, insectarium, photonic nanoarchitecture, population genetics, reflectance spectrum, structural colour

## Abstract

**Simple Summary:**

This study introduces a cost-effective and environmentally friendly laboratory procedure and infrastructure that can be readily applied to industrial production. We demonstrate that a custom-made insectarium can yield natural, finely tuned photonic nanostructure surfaces year-round using Common Blue butterfly laboratory populations. Using a single device, this process enables the production of more than 7500 wing samples annually, equivalent to up to 1 m^2^ of photonic surfaces. To ascertain the reliability of Common Blue as a source of photonic nanostructures, we compared the structural colour of the laboratory population to different natural genetic lineages. Although clear signs of genetic erosion due to inbreeding was detected through molecular genetic variability analysis, we observed minimal differences in the structural colouration showing the exceptional stability of the photonic nanostructures in the wing scales of Common Blue males. Consequently, there is a notable economic opportunity in developing technology that can be applied in industry to produce these natural materials that may be used directly in different kinds of applications requiring intricate photonic nanostructures.

**Abstract:**

The colour of the butterfly wing serves as an important sexual and species-specific signal. Some species produce structural colouration by developing wing scales with photonic nanoarchitectures. These nanostructures are highly conservative, allowing only a ±10 nm peak wavelength deviation in the reflectance spectra of the blue structural colour in natural Common Blue (*Polyommatus icarus*) populations. They are promising templates of future artificial photonic materials and can be used in potential applications, too. In this work, we present methodology and infrastructure for breeding laboratory populations of Common Blue as a cost-effective and environmentally friendly source of nanostructures. Our technology enables the production of approximately 7500 wing samples, equivalent to 0.5–1 m^2^ of photonic nanoarchitecture surface within a year in a single custom-made insectarium. To ascertain the reliability of this method, we compared reflectance properties between different populations from distant geographic locations. We also provide genetic background of these populations using microsatellite genotyping. The laboratory population showed genetic erosion, but even after four generations of inbreeding, only minimal shifts in the structural colouration were observed, indicating that wild Common Blue populations may be a reliable source of raw material for photonic surfaces.

## 1. Introduction

The search for technical solutions in the organic world has deep roots lost in the early times of human history. For example, almost all the cultures discovered that the scaled surface of various reptiles may work as a protective shield, hence the helms of the warriors mimicked crocodile or turtle armour, and as the applied technology advanced the material, they turned from animal skin to steel wire. Similarly, all the cultures discovered the efficient manner of pelt in thermal regulation, which was used for various isolation techniques, but finally was replaced by synthetic materials. Therefore, the bio-mediated structures are copious sources for the innovation of human technology [1].

Breeding of insects was always in the centre of interest for many human cultures. For example, as an important source of food supply, beekeeping is known all over the world; there are records from early historic times. Several European nations have long and distinctive traditions how to breed the domesticated honey bee *Apis mellifera* [2]. Similarly, many species of saturnid moths or pierid butterflies were harvested because their organic production was the basis of the silk necessary for the textile industry [3,4]. Nevertheless, the best example is the bombycid moth *Bombyx moori*, which became a domesticated animal during the millennia of silk manufacturing in Asia [5].

Apart from commercial production in butterfly farms [6], modern insect breeding in laboratories concentrates to solve working hypotheses generated by evolutionary scenarios or conservation purposes. For instance, extensive laboratory breeding programs have been undertaken in the family Lycaenidae. These aimed to map the genetic background of the phenotypic appearance of the *Aricia allous* superspecies [7], or to find the most efficient way to produce a large number of individuals for reintroduction of *Glaucopsyche lygdamus palosverdesensis* [8], or to record how *Polyommatus icarus* reacts to dietary changes [9] or cold stress [10,11,12], or how range expansion [13] and nuclear pollution [14] influenced *Zizeeria maha*, etc. However, none of these experiments have a strict applied aspect like the invention of silk production by mass rearing of *Bombyx mori* offered for textile industry [5].

Since it is possible to evaluate organic structures in nanoscale dimensions, science turned to these sources more intensively and the terms bioinspiration, biomimetics, biomorphy, bionics, and biotemplating appeared in the literature [15]. The diversity of these terms shows that although the efforts of modern technological approaches are intensive, they focus on the same starting point: biology. Besides the ethical questions, when nature protection laws hinder the inclusion of animals into various projects, another problem of bio-mediated research is that the available samples may have different biological origin or can be restricted in quantity. Seemingly, although collections of natural history museums could serve as a firm basis of samples for bioinspired studies, the reality is different: many of the museum specimens are unique samples; therefore, large series for statistical analysis are seldom available, and investigations have to be carried out in a non-destructive manner [16].

Working with butterflies with photonic nanoarchitectures in their wing scales with roles in sexual signalling [17,18,19,20] or camouflage [21,22], we faced the problem that the availability of museum samples is restricted, and in many interesting cases, destructive analyses are impossible. Moreover, the variables, which may influence the scale development and formation during the metamorphosis of the individual insect hitherto remain unexplored [18,23].

During our research, we discovered that the wings of the male Common Blue (*Polyommatus icarus*) grown in laboratory conditions are particularly suitable for experiments with photonic materials in which a large number of samples with the same optical properties are required. Due to the role played by the blue colour in sexual communication, the photonic nanoarchitectures that produce the colour show outstanding stability in space and time [24]. At the same time, in the case of butterfly specimens reared under controlled environmental conditions, in contrast to individuals collected in natural habitats, certain influencing variables are eliminated (time elapsed from eclosion to capture time, the effects of weather conditions, etc.); therefore, we can achieve a large increase in reproducibility and “productivity”. Breeding the Common Blue in laboratory requires significantly lower costs, chemicals, and highly skilled working hours than the production of inverse opal-type nanoarchitectures with the same optical properties [25], which would require an investment significantly higher in magnitude.

In this study, our objective is to introduce a comprehensive methodology and infrastructure for breeding the Common Blue butterfly species on a large scale, with the aim of harnessing its potential as a prolific source of photonic nanoarchitectures. We provide comprehensive details on the precise control and year-round maintenance of the breeding process, including guidelines for the optimal harvesting of butterfly wings with structural colours. We also describe the genetic structure of our laboratory population using microsatellite genotyping and compare it to native populations. Additionally, we elucidate the methodologies employed for the analysis of both optical and genetic properties of the obtained samples.

## 2. Materials and Methods

### 2.1. The Butterfly

The butterfly species *Polyommatus icarus* (Rottemburg, 1775), in vernacular English: Common Blue [26] is one of the most common butterfly species in Europe; it has a Transpalearctic distribution and very recently the Nearctic region has been colonized [27], too. The Common Blue is associated with natural and managed species-rich open grasslands in the Palearctic region (and in North America [27]), where its main larval hostplants, *Lotus corniculatus*, *Lotus uliginosus*, *Medicago lupulina*, *Ononis minuta*, and *Ononis spinosa* occur. Whilst species-rich grasslands have become scarcer because of agricultural intensification and change of land use, the species remains widespread as it also inhabits roadsides, brownfield sites, and semi-urban regions with open areas [28]. Consequently, the species is not subjected by any national or international nature conservation law. The species is genetically rather uniform on large territories in Europe: Dincă et al. [29] report, in a continental-scale phylogeographical analysis, a single genetic lineage (i.e., the northern Palearctic lineage) that dominates most of Europe. Although peripheral territories still host different lineages (see also Arif et al. [30]), our sampling has been confined to the central territory that corresponds with the area of the northern Palearctic lineage. Similar to the finding of Dincă et al. [29], our previous work [23] that used microsatellites has found only minute genetic differences between Western and Central-Eastern European populations.

### 2.2. Laboratory Population

In order to source photonic nanostructures for applications, we established a laboratory population of Common Blue from a native population in Hungary, near the settlement Érd (coordinates: 47°22′20.1″ N 18°54′12.5″ E). This population was haphazardly sampled by field capture using butterfly nets for three founder pairs of individuals (generation G_0_) on the 1st of October in 2019. These founders were kept in our insectarium (detailed technical description of the device can be found below) that—under controlled environmental conditions—gave rise to four subsequent generations. Each of these generations were inbred in the insectarium by selecting three pairs for breeding and returning to the device. The eclosion of the fourth generation (G_4_) ended on the 2nd of April in 2020. Spectral and population genetic analyses were conducted on the harvested specimens to investigate the effect of the potential genetic drift on structural colour.

Laboratory populations G_8_, G_10_, and G_11_ were first generation offspring of single breeding pairs originated from the same native population in Érd, Hungary as shown above.

Population G_4_ was investigated in the structural colour and population genetic analyses involving inbred specimens, whilst G_8_ was used to compare the variance of structural colour with natural populations monitored in a single day or through a whole year. Populations G_10_ and G_11_ were involved in the comparison of eclosion times in two consecutive breeding cycles.

Specimens used for comparison were captured in 2017–2018 from European populations in Taizé, France (coordinates: 46°30′06.7″ N 4°39′54.0″ E), in two very close locations in the region of Érd, Hungary (coordinates: 47°22’20.1″ N 18°54′12.5″ E and 47°22′08.3″ N 18°54’41.6” E), and in Barațcoș, Romania (coordinates: 46°38′22.5″ N 25°59′01.2″ E). The material is inventoried and deposited in the curated collection of the Centre for Energy Research, Institute of Technical Physics and Materials Science (Budapest, Hungary).

### 2.3. The Insectarium

**Figure 1:**
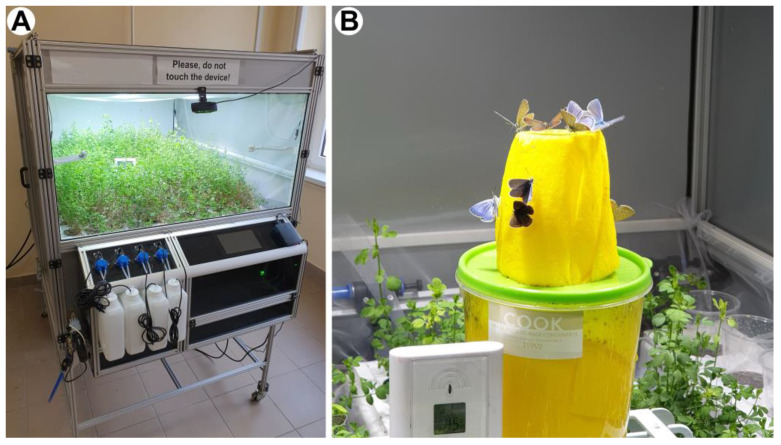
(**A**) A custom-made insectarium for the breeding of Common Blue (*Polyommatus icarus*) (dimensions: 120 cm × 100 cm × 60 cm). Inside the device, the host plant of the species, *Lotus corniculatus* was cultivated. (**B**) Freshly hatched imagines feeding on sugar solution.

A custom-made insectarium with a base area of 120 cm × 100 cm and height of 60 cm was developed to provide a 720-L flying arena. The climate conditions were carefully manipulated to replicate warm and extended summer days, creating an environment conducive to courtship and mating of *P. icarus* specimens (Figure 1). Using this device, it was possible to obtain consecutive generations all year, regardless of the conditions offered by the season. The illumination was provided for 14 h per day by seven 50 W white light LEDs, which also set the temperature around 30 °C inside the insectarium. The larval hostplant *Lotus corniculatus* was grown from seed using a six-channel hydroponic plant growing system, which was the basis of the device, in which 72 stems were placed in six rows (Supplementary Video S1). The larvae were fed on *L. corniculatus* buds and leaves, while the imagines were fed with sugar solution which was stored in a feeder that was placed in the centre of the flying arena. The 1L tank was filled with the mix of sugar and water, and a piece of fabric that mimicked the colour of the host plants’ bright yellow flowers absorbed this solution, providing a continuous source of nutrient; therefore, the imagines were able to drink on the whole surface of the feeder (Figure 1). After eclosion, the butterfly specimens were harvested, frozen, and placed individually in small plastic containers (Ted Pella, Inc., Redding, CA, USA), then inventoried. The whole material was stored in large plastic boxes in a refrigerator at −18 °C to avoid pests.

### 2.4. Reflectance Spectrophotometry

Reflectance spectroscopy measurements were conducted on Common Blue specimens using an Avantes (Avantes BV, Apeldoorn, The Netherlands) modular fibre-optic system consisting of an AvaSpec-HERO spectrophotometer, an AvaLight-DH-S-BAL stabilized UV-visible light source, an AvaSphere-30-REFL integrating sphere, and a bifurcated reflectance probe (FCR-7UV200-ME-SR). For integrating sphere measurement mode, a WS-2 white diffuse tile was used as a reference.

The plotting and the statistical analysis of the reflectance data were carried out using Origin 2021 (OriginLab, Northampton, MA, USA) software (version 9.8.0.200). The peak wavelength and amplitude data of the samples were evaluated using one-way ANOVA followed by Tukey’s post hoc test (*p* < 0.05) for all pairwise comparison procedures.

### 2.5. Population Genetics

In order to characterize the genetic background of our laboratory population (G_4_), we genotyped them for the microsatellite loci developed by our team [23]. We used 18 single sequence repeats (SSRs) regions for the evaluation of the population genetic characteristics of this population and three additional populations sampled previously [23]: the mother population of our G_4_ population in Érd (Hungary), and two geographically distinct populations in Taizé (Burgundy region, France) and in Baraţcoş (Ghimeş region, Romania). The DNA extraction procedure, microsatellite marker development, and evaluation followed the protocol described earlier [23]. Briefly, raw reads were imported into PeakScanner v.1.0 (Applied Biosystems, Waltham, MA, USA) and genotypes were generated by examining the electropherograms. Genotypes were entered into Excel (Microsoft Inc., Redmond, WA, USA) then analysed by GenAlEx v.6.5 [31], which was used for calculating basic allelic statistics and the unbiased heterozygosity (i.e., genetic diversity) for each population, and the pairwise unbiased Nei’s genetic differentiation (G_st_) and its visual representation by standard principal coordinate analysis (PCoA). Allelic frequencies for each locus per population were imported into PAST v.3.04 software [32] to unravel the genetic relationship between the studied populations by means of unweighted pair group method with arithmetic mean (UPGMA) tree building. This tree was constructed by first calculating Cavalli-Sforza chord distance based on raw allelic frequencies then, using the paired-group algorithm, building the dendrogram with statistical test of branch robustness using 1000 non-parametric bootstrap. Finally, the dataset was imported into R [33] by the read.genalex function of the package poppr v. 2.9.3 [34], and the Provesti-distance was calculated for each sample using the function provesti.dist. This distance matrix was used to reconstruct the possible phylogenetic relationship between the samples by turning the distance matrix into a bioNJ (neighbor-joining) dendrogram using the aboot function of the above package.

## 3. Results and Discussion

### 3.1. Breeding

Common Blue specimens were reared under controlled conditions in a custom-made insectarium (Figure 1). Previously, a breeding experiment was carried out in outdoor conditions (Figure S1) [11], but in that case we had to face similar difficulties that affect natural populations: due to the changing light and climatic conditions through the year, only a limited number of butterflies were hatched in the summer in a maximum of three consecutive generations. At the same time, eggs, larvae, and imagines may all have experienced external influences, such as predators in the vegetation or extremities in weather which had an impact on the breeding output.

**Figure 2:**
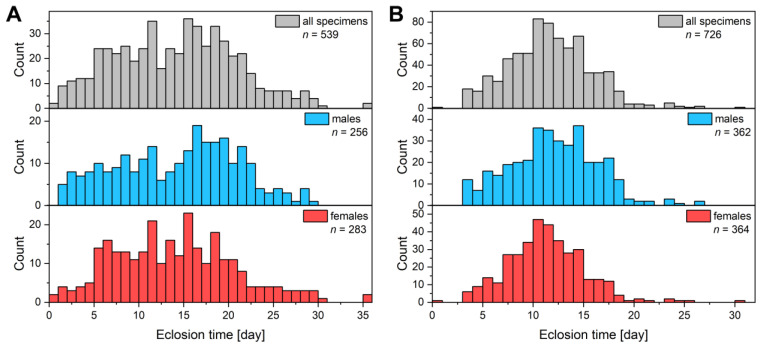
Eclosion histograms of two consecutive breeding cycles. (**A**) Generation G_10_ (*n* = 539) and (**B**) G_11_ (*n* = 726) are shown. The start of the eclosion time is taken the day when the first imagine was observed in the insectarium. Males and females follow similar patterns that result in almost 50–50% proportion of the sexes.

**Figure 3:**
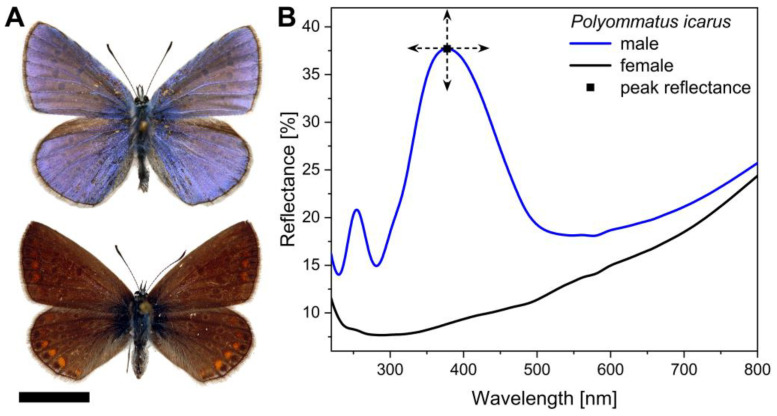
(**A**) Photographs of male (**top**) and female (**bottom**) set museum specimens of Common Blue (*Polyommatus icarus*) in dorsal view (Scale bar: 1 cm). (**B**) The corresponding reflectance spectra of both sexes are shown: the structural blue colour of the male has a characteristic peak at around 400 nm, while the brown female lacks this. The arrows illustrate the possible peak wavelength and peak amplitude variations (not to scale) can be measured between the different specimens.

In contrast, due to the strictly controlled environment, we successfully replicated this reproduction cycle, ensuring a consistent and steady production rate. The laboratory populations were able to reproduce throughout the whole year without overwintering. As a result of the favourable conditions, the entire lifecycle of the species was completed exclusively inside the insectarium: the collected wild pairs of Common Blue mated, the females laid eggs, the eggs were hatched, then the larvae fed on *L. corniculatus* leaves. After an intensive growth period and four moulting stages, the larvae successfully pupated. The hatched butterflies were harvested from the device as soon as they dried their wings and started flying to preserve their initial pristine condition. One cycle of breeding usually lasted 7–8 weeks, which is similar to the duration that was measured for natural populations [17] and in the previous outdoor breeding experiments [11]. The main difference was that in the laboratory population, all larvae skipped diapause; therefore, we were able to repeat the reproduction cycle of the butterflies roughly every two months. During one cycle of breeding, we managed to breed 500 to 700 specimens from a single pair; thus, we achieved a huge increase in “productivity” compared to the outdoor breeding. Furthermore, samples could also be reared in the winter, when there was neither suitable vegetation nor imagines in the wild. The proportion of males and females were almost equal (Figure 2), which thus enables the large-scale production of specimens with blue structural colour. Considering that the typical dorsal wing surface area of a male specimen is around 3–4 cm^2^ (Figure 3A), it is possible to generate a total of 0.5–1 m^2^ of photonic nanoarchitecture surfaces in a single insectarium over the course of a year.

### 3.2. Spectral Properties

An important question is whether the breeding process in a controlled environment affects the blue colour of the butterflies. The structural colour of males in polyommatine blues is originated from the dorsal cover scales with the interplay of pigments and photonic nanoarchitectures, whilst the females are generally brown (Figure 3A) [35] except at the edges of the species’ range where they may be blue [36,37,38,39]. The photonic nanoarchitectures are composite materials in which the refractive indices of the transparent components (chitin and air) are different enough, while their spatial periodicity is comparable with the wavelength range of visible light to form a photonic band gap [40,41]. This means that light with a certain wavelength range cannot propagate in this nanocomposite and, therefore, is selectively reflected from its surface so that the eyes of the observer can perceive it as structural colour, which can be measured with a spectrophotometer in form of a reflectance spectrum (Figure 3B). The light passing through/scattered by the nanoarchitecture is absorbed by broadband absorber pigments, such as melanin in this case [42].

Previously, we have found that the structural colours of polyommatine lycaenid butterflies that are generated by “pepper-pot”-type photonic nanoarchitectures have an important role in species discrimination occurring in the prezygotic sexual communication of these insects [35], which strongly limits the range of colours suitable for mating; therefore, only species-specific colours are applicable with success [17]. Consequently, the structural colours are exceptionally stable: the variation of the spectral position of the reflectance maximum falls within a ±10 nm wavelength range for males collected from a local population at the same day [18]. They are also very similar within Europe [23,24] and also remained unchanged over 100 years when museum specimens were investigated [24], meaning that strong selective pressure led to an accurate reproduction of photonic nanoarchitectures with nanometre precision even under natural conditions. This natural variation can be further reduced in the case of specimens reared in laboratory conditions. In this study, the caterpillars were fed upon the same plants; there is certainly no variance in the nutrients they take in [43], and since they descended from a single or three breeding pairs, their genetic variability can be much smaller than in the natural populations.

**Figure 4:**
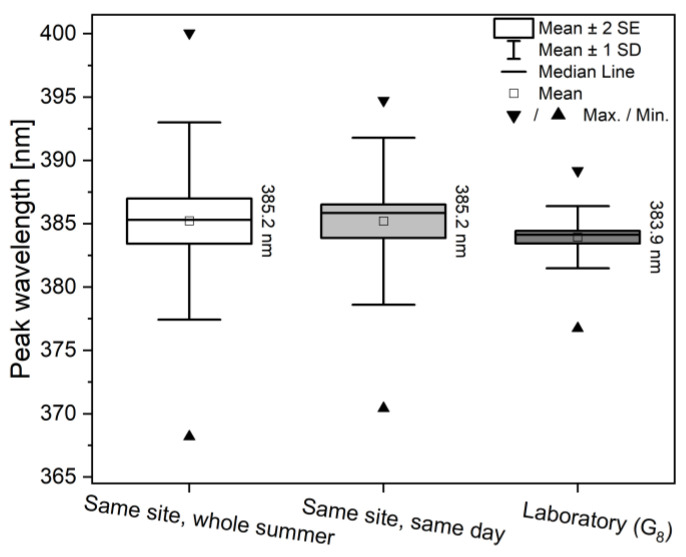
Box-plot representation of the peak wavelength variation of male Common Blue (*Polyommatus icarus*) specimens from different collecting conditions. A sampling site near Budapest was monitored through a year (*n* = 76), and it was compared to specimens collected on a single day at the same site (*n* = 100) and to a laboratory population with the same origin (G_8_, *n* = 100). The average peak wavelengths are almost identical, while the variation greatly decreases with the reduction of environmental variables. There were no statistically significant differences between the means of the three populations (*p* < 0.05).

The comparison of spectral variation of the different populations (Figure 4) shows that the standard deviation of the laboratory specimens (four wings of 25 specimens: *n* = 100) resulted in the first generation offspring of a single breeding pair (G_8_) being much smaller compared to specimens originating from a natural habitat sampled for a single day (*n* = 100) or a whole year (*n* = 76), suggesting that when fewer variables are allowed to change, the smaller deviations will be present and more uniform structural colours can be obtained. The means of the peak wavelength showed no significant differences between the three populations using one-way ANOVA (*p* < 0.05). This effective reproduction can also play an important role in the utilization of butterfly wings, since a structural colour with a small standard deviation also means a high-precision photonic nanoarchitecture generating it, which thus can be used directly as a ready-made prototype in a potential application that requires highly similar samples, such as vapor sensing [44,45,46,47], biotemplated photocatalysis [48,49,50], or surface-enhanced Raman spectroscopy [51,52].

**Figure 5:**
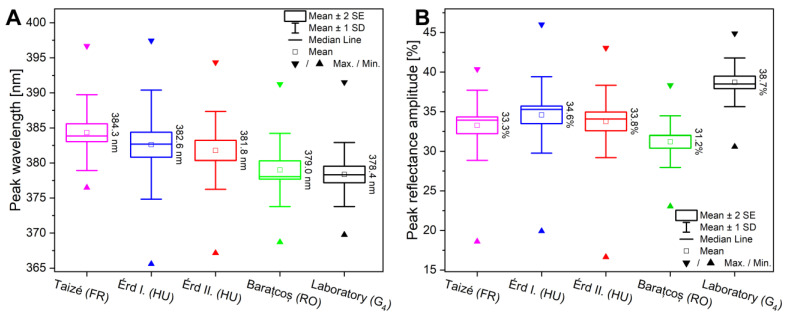
Box-plot representations of the (**A**) peak wavelength and (**B**) the peak reflectance amplitude data of the blue structural colour of male Common Blue specimens. The three European sampling sites from Taizé, France; Érd, Hungary; and Baraţcoş, Romania were compared to laboratory population (G_4_), which was reared in the custom-made insectarium. One-way ANOVA showed statistically significant differences between the peak amplitude means of the populations supplemented with post hoc Tukey’s test (*p* < 0.05).

The structural colour diversity of laboratory specimens was compared to natural European populations, and the possible correlation with their population genetic structure was investigated. For this purpose, we measured the reflectance of the four wings of 20–20 specimens from four sampling sites in nature together with the 20 specimens from a laboratory population. We found that the structural colour mean of the laboratory population (G_4_) had a minor shift from the natural populations, but their average peak wavelength fell in the ±10 nm wavelength range, typical of the European specimens (Figure 5A) [24]. One-way ANOVA showed statistically significant differences between the peak wavelength means (*p* < 0.05), and with post hoc Tukey’s test, pairwise differences were found between the laboratory population (G_4_) and all Hungarian and French populations, while the Romanian and the laboratory populations were not statistically different. In contrast, the laboratory population had more intense structural colour, which was shown by the higher average peak amplitude value of 38.7% (Figure 5B) compared to the others. It was also confirmed by the statistical analysis as significant pairwise differences were found between the peak amplitude means of the laboratory (G_4_) and all other populations (*p* < 0.05). This is due to the fact that the laboratory specimens were captured from the insectarium as soon as they started flying, thus preserving their initial, pristine condition, while specimens from natural populations may have been in their habitat for days before capture under variable conditions and influences, which affected the number of structurally coloured scales covering their wings and thus the amplitude of the reflectance peak.

### 3.3. Population Genetics

**Figure 6:**
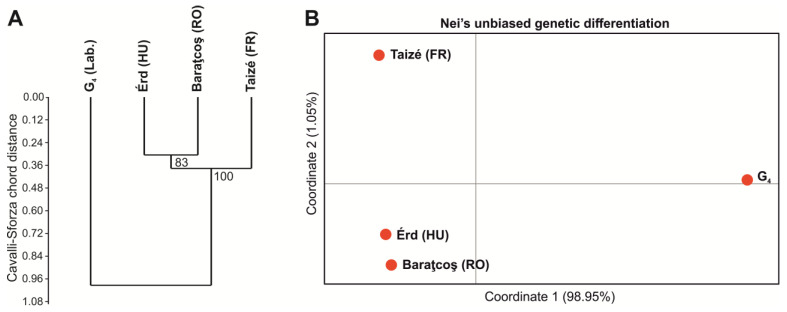
(**A**) Genetic distance and (**B**) genetic differentiation between the investigated populations of Common Blue butterflies using 18 nuclear microsatellites. (**A**) Unweighted pair group method with arithmetic mean (UPGMA) dendrogram constructed from Cavalli-Sforza chord distance based on raw allelic frequencies of the populations. The numbers are branch support values resulting from 1000 non-parametric bootstrap. (**B**) Principal coordinate analysis (PCoA) ordination along the first two axes based on Nei’s unbiased measure of genetic differentiation (G_st_).

The population genetic structure of the four groups was investigated. Although nuclear microsatellites or single sequence repeats (SSRs) are in the non-coding part of the DNA, as they are known to have exceptionally high mutation rate [53], they are a good choice for tracing the neutral genetic variability of natural populations. The genetic relationship between our studied populations (i.e., the laboratory population and three natural populations from different parts of Europe) based on raw allelic frequencies show substantial separation of the laboratory population from the natural populations (Figure 6A). If we only focus on the natural populations, their relationship reflects geographical distance: the Hungarian and the Romanian populations are closer to each other, whereas the French population is more distant.

Similarly, genetic differentiation is remarkable (Figure 6B): the populations separate along the first axis that already covers 98.95% of the variance, and it separates the laboratory population (G_4_) from the rest. Between the natural populations, the difference is apparently proportional to their geographic distance. In terms of absolute values, mean G_st_ between the natural populations was only 0.037, whereas the differentiation of the laboratory population from the natural ones was 0.2234.

This high level of genetic differentiation in the laboratory population must be associated with the combined effect of (i) using three pairs as founders of the captive population (founder effect) and (ii) four generations of inbreeding. These two factors led to severe genetic drift in the laboratory population compared to the natural ones. The above process is further demonstrated by the genetic diversity values (Table 1): the laboratory population shows a significant drop in genetic diversity and high levels of inbreeding, which is fully compatible with a population history affected by the above-mentioned population genetic mechanisms. In spite of this strong reduction in population genetic characteristics, the structural colour of the investigated specimens was almost unchanged (Figure 5), which indicates a robust genetic background of nanostructure formation that was not affected by the genetic erosion resulting from the strong founder effect and four generations of inbreeding.

**Figure 7:**
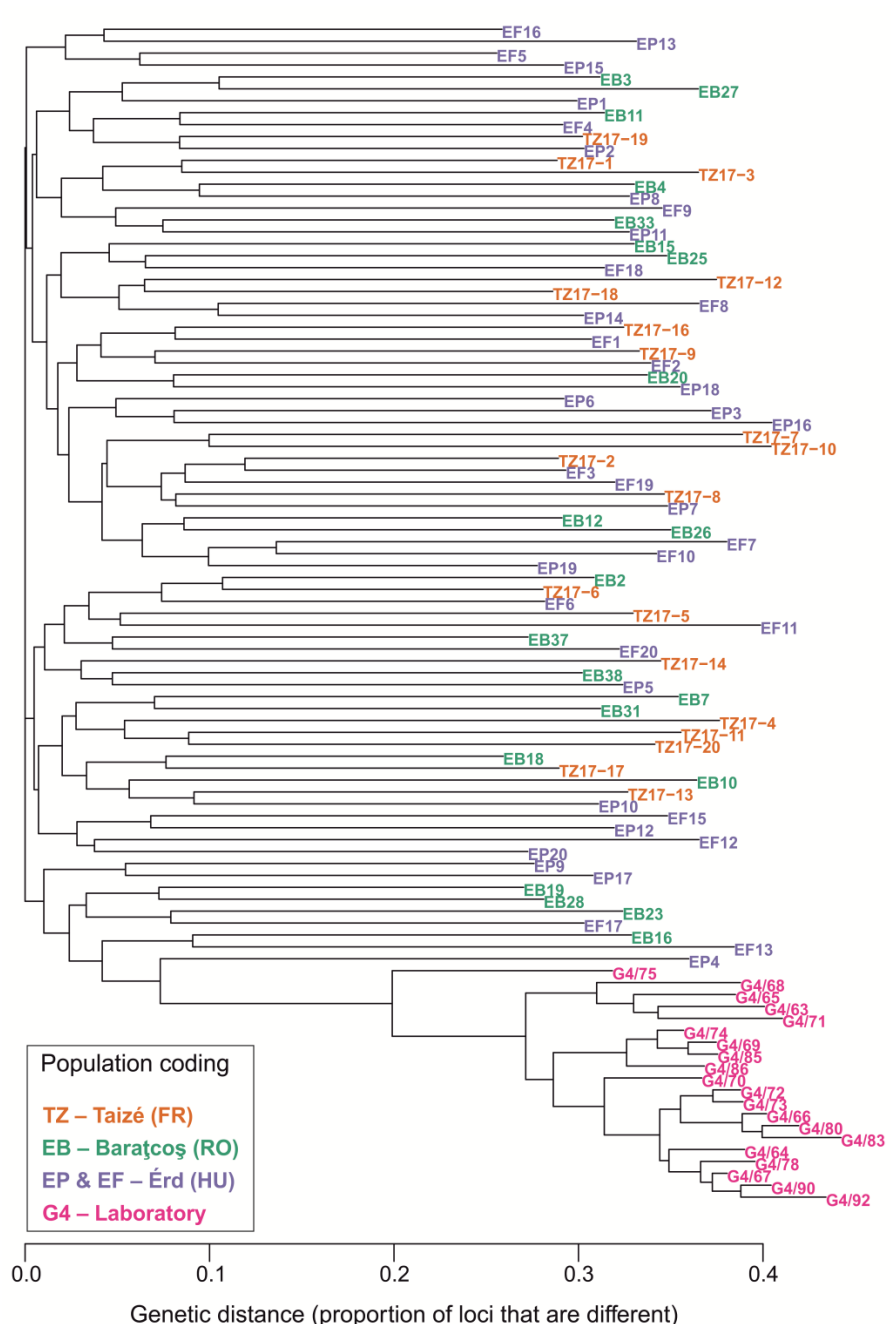
Phylogenetic origin of the laboratory population as represented by a bioNJ dendrogram constructed from a genetic distance matrix between the studied individuals based on Provesti-distances. The individual samples are colour coded according to population.

In contrast to the population-level analyses, the phylogenetic origin of the laboratory population (as represented by the individual-level genetic distance based on Provesti-distances) remained unaffected (Figure 7). The laboratory population forms a well-separated monophyletic cluster, and its direct potential sister lineage is a sample from the Érd population (namely EP4). Similarly, the monophyletic branch in which the laboratory lineage is nested also contains only samples from the mother population (Érd) and some samples from the genetically closely related (Figure 6B) Baraţcoş population.

In summary, our laboratory population that was kept for four generations in captivity shows all population genetic characteristics described for small, inbred populations usually studied in the context of conservation [54]. However, this impoverishment in neutral genetic characteristics has not influenced photonic nanostructures and, thus, cannot impede the industrial use of our technology detailed below.

### 3.4. Biotemplating for Industrial Applications

As we reported recently in several papers, various butterfly wings possessing structural colour were successfully used as biotemplates with complex nanoarchitecture for the deposition of thin films with photocatalytic properties [49,50]. The use of butterfly wings for building hybrid photocatalytic platforms based on biological architectures with complex, hierarchic structures from the nanometre to centimetre scale opens a wide route to combine various photocatalytic nanoparticles with thin films conformally deposited by atomic layer deposition that allows spectral engineering of the optical properties of the photocatalyst in order to tune them to the specific absorption of the substances to be eliminated [55,56]. All over the world, numerous butterfly families possess intense colours of structural origin; for example, we recently investigated several species from the genus *Arhopala* that covers the spectral range from the UV to green [19]. In our work focusing on *Morpho* butterflies, we were able to increase the catalytic efficiency of the butterfly wings covered by a conformal 20 nm thin layer of ZnO by about ten times as compared to a flat glass substrate [50]. Previous work on plasmonic Au nanoparticles and the photonic nanoarchitectures of male *Polyommatus icarus* wings showed that the Au nanoparticles incorporated into the photonic nanoarchitecture of the butterfly wings exhibited hybridization between the photonic nanoarchitecture and the metallic nanoparticles with plasmonic properties [57]. These findings strongly support our belief that combining photonic nanoarchitectures of biologic origin with various well established materials science methods may bring important advantages in a cheaper and more environmentally friendly way in several fields.

## 4. Conclusions

The Common Blue can be turned into a laboratory insect to produce high-quality photonic nanoarchitectures, as a large number of samples with the same optical properties can be achieved which are not sensitive to inbreeding. The reproducibility of the structure and colour of such nanoarchitectures might be governed by a precise, genetically controlled self-assembly mechanism of scale growth. At the same time, the process of obtaining the well-reproduced photonic nanoarchitectures is based on the breeding of herbivorous insects, which does not raise environmental concerns and needs only moderate amounts of energy input and work hours of highly qualified operators. We demonstrated using our custom-made insectarium that ready-made prototypes of finely tuned photonic nanostructure surfaces can be yielded year-round using Common Blue laboratory populations in a cost-effective and environmentally friendly manner. Our breeding system is scalable. Using a single insectarium, we were able to breed 500–700 butterflies every two months, which in summary, can result in approximately 7500 optically highly similar wing samples together constructing 0.5–1 m^2^ of structurally blue coloured surface annually that may be used directly in different kinds of applications requiring intricate photonic nanoarchitectures.

## Figures and Tables

**Table 1:** Basic population genetic characteristics of the studied *Polyommatus icarus* populations as reported by GenAlEx (mean ± s.e.). Na—number of different alleles, Ho—observed heterozygosity, He—expected heterozygosity, uHe—genetic diversity, Fis—fixation index.

Population	Na	Ho	He	uHe	Fis
Baraţcoş (RO)	7.75 ± 0.864	0.617 ± 0.068	0.675 ± 0.064	0.692 ± 0.066	0.099 ± 0.039
Taizé (FR)	7.813 ± 0.918	0.557 ± 0.05	0.682 ± 0.054	0.701 ± 0.055	0.164 ± 0.046
Érd (HU)	9.688 ± 0.869	0.556 ± 0.05	0.682 ± 0.058	0.692 ± 0.059	0.179 ± 0.03
Laboratory (G_4_)	1.813 ± 0.164	0.361 ± 0.083	0.253 ± 0.052	0.26 ± 0.053	−0.363 ± 0.063

## Data Availability

All relevant data analysed during this study are included in this published article and its Supplementary Information Files. Raw datasets used during the current study are available from the corresponding author on reasonable request.

## Supplementary Materials

The following supporting information can be downloaded at: https://www.mdpi.com/article/10.3390/insects14080716/s1, Figure S1: Outdoor breeding setup for Common Blue (*Polyommatus icarus*) butterflies; Video S1: Timelapse video from the inside of the insectarium recorded from 11 December 2019 to 20 December 2019.
