# Supplementary material for: Breeding Polyommatus icarus Serves as a Large-Scale and Environmentally Friendly Source of Precisely Tuned Photonic Nanoarchitectures
Source: Insects. 2023 Aug 18;14(8):716. doi: 10.3390/insects14080716 (PMC10455773; doi:10.3390/insects14080716)
Supplement: Supplementary file 1 [file insects-14-00716-s001.zip › Supplementary figure SI.pdf]

Supplementary Information

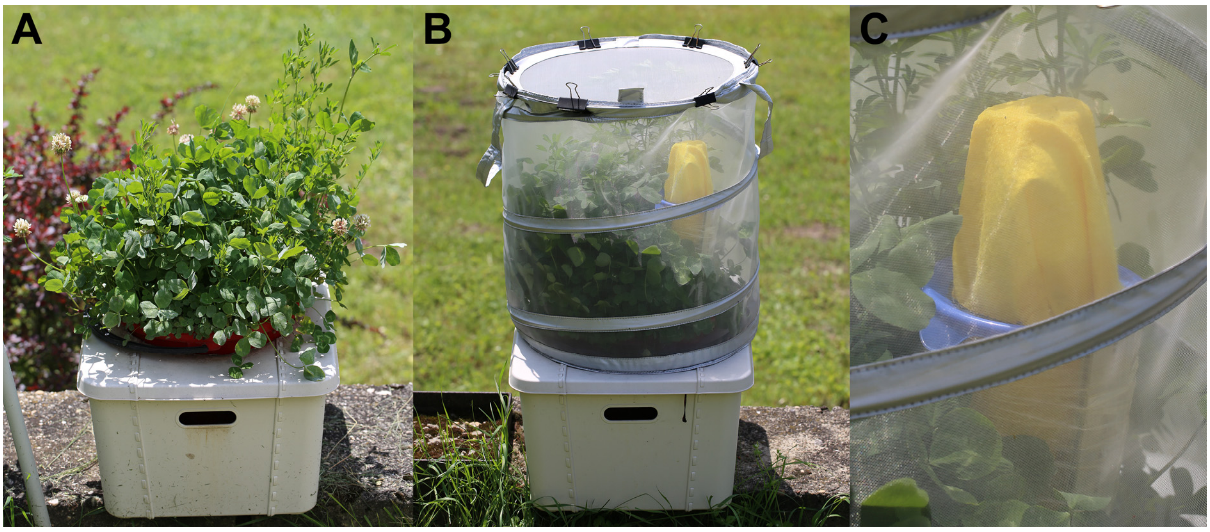

Figure S1. Outdoor breeding setup for Common Blue (*Polyommatus icarus*) butterflies. (**A**) Hostplants of the species *Trifolium repens*, *T. pratense* and *Medicago sativa* were cultivated. (**B**) These were covered with an openable net, (**C**) and a feeder was also included in the middle, which contained sugar water and mimicked the yellow color of the flowers of *Lotus corniculatus*.

Video S1. Timelapse video from the inside of the insectarium recorded from 11th December 2019 to 20th December 2019. Still photographs were taken every 10 minutes during the day and stitched together into a 30-fps video. The rapid growth of *Lotus corniculatus* and the flying imagines of newly hatched *Polyommatus icarus* can be seen.
